# Predictive efficacy of color Doppler in wrist radio-cephalic AV fistula creation: A prospective study

**DOI:** 10.6026/973206300212417

**Published:** 2025-08-31

**Authors:** Shashi Kumar, Nallabothula Anil Kumar, Chilekampalli Konda Reddy, Nalumaru Anirudh Suseel

**Affiliations:** 1 Department of Urology, Sri Venkateswara Institute of Medical Sciences, Tirupati, Andhra Pradesh, India

**Keywords:** Color Doppler, radio-cephalic arteriovenous fistula, hemodialysis access, fistula maturation, vascular ultrasound, preoperative assessment

## Abstract

High rates of early failure and non-maturation remain major challenges in wrist radio-cephalic arteriovenous fistula (RC-AVF) creation for hemodialysis access. Therefore, it is of interest to assess the role of preoperative color Doppler ultrasonography (CDUS) in predicting successful fistula maturation in 80 patients. Radial artery diameter ≥2.0 mm and cephalic vein diameter ≥2.5 mm were significantly associated with maturation (p < 0.05), with CDUS showing 87% sensitivity and 75% specificity. Higher flow volumes also correlated with better outcomes. Preoperative CDUS is a valuable tool that enhances patient selection and improves AVF success rates.

## Background:

Chronic kidney disease (CKD) continues to be a significant global health concern, with hemodialysis being the most widely used renal replacement therapy in end-stage renal disease (ESRD) patients [1]. Establishing reliable vascular access is critical for effective hemodialysis and among the available options; the radio-cephalic arteriovenous fistula (RC-AVF) remains the preferred choice due to its lower complication rates and longer patency [2]. However, despite being the first-line access, RC-AVFs are associated with a notable incidence of early failure and non-maturation, leading to increased morbidity, hospitalization and costs [3, 4]. To mitigate the risk of AVF failure, preoperative vascular assessment has gained attention. Color Doppler ultrasonography (CDUS) is a non-invasive, widely available, and cost-effective imaging modality that provides detailed information about vessel diameter, wall condition and flow characteristics [5]. Several studies have highlighted the predictive value of CDUS parameters such as radial artery diameter, cephalic vein diameter and arterial flow volume in determining the likelihood of successful AVF maturation [6, 7]. The National Kidney Foundation's Kidney Disease Outcomes Quality Initiative (KDOQI) recommends preoperative vascular mapping with Doppler ultrasound to guide AVF creation and enhance success rates [8]. However, the threshold values for vessel diameters and flow velocities that predict fistula maturation vary across studies and populations [9]. Hence, further investigation is warranted to establish reliable, site-specific predictive parameters. Therefore, it is of interest to evaluate the role of preoperative CDUS in predicting the functional outcome of RC-AVF creation in patients undergoing hemodialysis, with a focus on identifying key vascular parameters that influence fistula maturation.

## Materials and Methods:

This prospective, observational study was carried out over a period of 12 months from January 2020 to December 2021, at Department of Urology, Sri Venkateswara Institute of Medical Sciences, Tirupati and Andhra Pradesh, India. A total of 80 patients diagnosed with end-stage renal disease (ESRD) and scheduled for primary radio-cephalic arteriovenous fistula (RC-AVF) creation at the wrist were enrolled. Prior ethical clearance was obtained from the institutional ethics committee, and informed written consent was taken from all participants.

## Inclusion criteria:

Patients aged 18 to 75 years with ESRD who were suitable candidates for wrist RC-AVF based on clinical assessment and willing to undergo preoperative color Doppler evaluation were included.

## Exclusion criteria:

Patients with previous AVF on the same limb, significant vascular disease, upper limb edema, central venous catheter placement on the ipsilateral side, or unfit for surgery were excluded.

## Preoperative assessment:

All enrolled patients underwent thorough clinical evaluation and color Doppler ultrasonography (CDUS) of the upper limb arteries and veins. The Doppler study was performed using a high-frequency linear transducer (7-12 MHz).

## Key parameters assessed included:

[1] Radial artery diameter

[2] Cephalic vein diameter (both superficial and with tourniquet)

[3] Arterial flow velocity and volume

[4] Vessel wall characteristics and compressibility

Patients with radial artery diameter ≥2.0 mm and cephalic vein diameter ≥2.5 mm were considered ideal candidates for RC-AVF creation.

## Surgical procedure:

All fistulas were created by a single experienced vascular surgeon using a standard end-to-side anastomosis technique under local anesthesia. Postoperative care included regular monitoring for patency, limb positioning and hand exercises to promote maturation.

## Follow-up and outcome assessment:

Patients were followed up at 6 weeks postoperatively. Fistula maturation was assessed clinically (presence of thrill and bruit) and confirmed by repeat Doppler ultrasonography. Maturation was defined as a flow volume >600 mL/min and vein diameter >6 mm, suitable for two-needle dialysis access.

## Statistical analysis:

Data were analyzed using SPSS software version 25. Continuous variables were expressed as mean ± standard deviation and categorical data as percentages. Chi-square test and unpaired t-test were used to compare variables between matured and non-matured groups. A p-value <0.05 was considered statistically significant.

## Results:

A total of 80 patients underwent wrist radio-cephalic arteriovenous fistula (RC-AVF) creation during the study period. The mean age of the study population was 54.3 ± 11.2 years, with a male predominance (62.5%). Among the 80 patients, 64 (80%) achieved successful fistula maturation at 6 weeks, while 16 (20%) did not.

## Demographic and clinical characteristics:

The demographic profile and comorbidities of the study participants are summarized in Table 1 (see PDF). The majority of the patients were male (n=50, 62.5%) and hypertensive (n=56, 70%). Diabetes mellitus was present in 30% of the cases.

## Preoperative Doppler parameters:

Color Doppler findings revealed that the mean radial artery diameter in the matured group was significantly higher (2.3 ± 0.4 mm) compared to the non-matured group (1.8 ± 0.3 mm, p < 0.01). Similarly, cephalic vein diameter with tourniquet was greater in the matured group (2.9 ± 0.5 mm) than the non-matured group (2.3 ± 0.4 mm, p = 0.02). Preoperative flow volume also showed a statistically significant difference (Table 2 - see PDF).

## Fistula maturation outcome:

Of the 64 patients with matured AVFs, all demonstrated adequate thrill and bruit on clinical examination and fulfilled the ultrasound criteria. The 16 patients with failed maturation were either due to thrombosis, insufficient venous diameter, or poor flow volume.

## Predictive value of Doppler parameters:

Receiver operating characteristic (ROC) curve analysis showed that a radial artery diameter ≥2.0 mm had a sensitivity of 87% and specificity of 75% in predicting successful maturation. Similarly, cephalic vein diameter ≥2.5 mm had a sensitivity of 81% and specificity of 70% (Table 3 - see PDF). These findings indicate that preoperative Doppler assessment is highly useful in selecting appropriate patients for RC-AVF creation and in predicting successful outcomes (Tables 2 - see PDF and 3 - see PDF).

## Discussion:

Arteriovenous fistula (AVF) remains the preferred vascular access for hemodialysis due to its superior long-term patency and lower complication rates compared to arteriovenous grafts and central venous catheters [1, 2]. However, early failure and non-maturation continue to pose significant challenges, affecting up to 20-50% of RC-AVFs in various clinical settings [3, 4]. This prospective study demonstrated that preoperative color Doppler ultrasonography (CDUS) provides valuable predictive information regarding the success of wrist RC-AVF creation. Our findings showed that patients with a radial artery diameter of ≥2.0 mm and cephalic vein diameter ≥2.5 mm were more likely to achieve fistula maturation, aligning with previous studies that identified these cut-off values as reliable predictors [5, 6]. The positive correlation between vessel diameter and maturation emphasizes the need for thorough vascular mapping before AVF surgery [7]. According to Silva *et al.*, preoperative assessment with Doppler significantly improves maturation rates by enabling appropriate patient selection [8]. Flow volume was also a significant parameter in our study, with matured AVFs having a mean preoperative flow of 720 mL/min, compared to 520 mL/min in non-matured cases. Similar observations were reported by Malovrh, who highlighted the role of high-flow states in early AVF success [9]. The KDOQI guidelines also recommend a minimum flow volume of 500-600 mL/min as a target for successful dialysis access [10]. In the present study, CDUS achieved a sensitivity of 87% and specificity of 75% for predicting maturation when using radial artery diameter ≥2.0 mm as a threshold. This is comparable to the results reported by Wong *et al.*, who observed 85% sensitivity and 72% specificity using similar criteria [11]. Our ROC analysis reinforces the role of CDUS as a valuable screening tool, particularly in resource-limited settings where surgical revision options are limited. Strength of CDUS is its ability to detect subclinical vessel abnormalities such as calcification, thrombosis, or stenosis that may not be evident on clinical examination alone [12]. Hence, reliance on clinical assessment alone may be insufficient, especially in diabetic or elderly populations with compromised vascular anatomy [13].

Color Doppler and duplex ultrasound assessments-specifically evaluating vessel diameter, distensibility, and peak systolic velocity-play a critical role in predicting the maturation and success of wrist arteriovenous fistula creation, which is essential for optimal vascular access in chronic kidney disease patients undergoing hemodialysis [14, 15]. While our findings strongly support the role of CDUS in enhancing surgical outcomes, certain limitations must be acknowledged. The study was conducted in a single center with a relatively small sample size, which may limit generalizability. In addition, long-term patency rates and secondary intervention data were not included in the current analysis. Nevertheless, our results provide robust evidence that CDUS should be considered a standard component of preoperative evaluation before RC-AVF creation. Future multicenter studies with larger cohorts and long-term follow-up are warranted to validate these findings and optimize Doppler-based criteria for AVF planning. In addition to vascular diameter and flow measurements, vein compressibility and wall thickness also influence fistula success. In our study, non-matured AVFs frequently demonstrated poor vein compressibility or increased wall echogenicity during preoperative Doppler examination. Such structural alterations are often found in patients with previous intravenous cannulations or longstanding comorbidities such as diabetes and hypertension, both of which can impair venous elasticity and adaptability to increased hemodynamic stress [6, 7]. This underlines the importance of a comprehensive CDUS evaluation, not limited to luminal diameter alone, but also including dynamic vessel properties. Furthermore, the skill of the operating surgeon, technique used (end-to-side vs. side-to-side anastomosis), and postoperative care also significantly affect fistula outcomes. While these variables were standardized as much as possible in this study, inter-operator variability in real-world clinical practice can impact AVF maturation.

## Conclusion:

Preoperative color Doppler ultrasonography plays a crucial role in predicting the outcome of wrist radio-cephalic AVF creation. Parameters such as vessel diameter, flow volume, and compressibility provide valuable guidance for access planning. Incorporating CDUS into routine preoperative assessment can improve fistula maturation rates, reduce early failures, and enhance overall dialysis outcomes.
